# Dowling-Degos-Like Flexural Hyperpigmentation in a Two-Year-Old Saudi Girl

**DOI:** 10.7759/cureus.109310

**Published:** 2026-05-20

**Authors:** Mona A Alshehri, Maram Alzain, Luluah Al-Mubarak, Lama M Almueqly, Raghad Almutairi

**Affiliations:** 1 Dermatology, Prince Sultan Military Medical City, Riyadh, SAU

**Keywords:** dowling-degos-like, flexural hyperpigmentaionn, hyperpigmentation, pediatric, reticulate hyperpigmentation

## Abstract

Dowling-Degos disease (DDD) is a rare, autosomal dominant pigmentary disorder typically characterized by adult-onset reticulate hyperpigmentation in flexural areas. Early-onset cases are exceptionally rare.

A two-year-old Saudi girl presented with a one-year history of progressive, asymptomatic, velvety hyperpigmented plaques involving the axillae, anogenital region, and face. Histopathological analysis showed epidermal acanthosis with elongated rete ridges and prominent basal hyperpigmentation, consistent with DDD. Immunohistochemistry (SOX10) confirmed increased melanocytes at the rete ridge tips.

This report describes what is, to our knowledge, the earliest documented case of DDD-like pigmentation in a patient under three years of age. It underscores the vital role of histopathological correlation in diagnosing pediatric pigmentary disorders, especially when genetic evaluations are inconclusive, and highlights the need for further research into the genotype-phenotype variations of early-onset disease.

## Introduction

Dowling-Degos disease (DDD) is a rare autosomal dominant pigmentary disorder characterized by progressive acquired reticulate hyperpigmentation, mainly involving flexural areas. It is associated with mutations affecting keratinization and melanosome trafficking pathways, most commonly involving KRT5, with additional pathogenic variants reported in protein O-fucosyltransferase 1 (POFUT1), protein O-glucosyltransferase 1 (POGLUT1), and presenilin enhancer 2 (PSENEN) [1-3]. DDD typically presents after puberty or in adulthood, and pediatric and early-onset presentations remain exceptionally uncommon [1,2]. Although prevalence data are limited due to the rarity of the disease, DDD is considered an uncommon genodermatosis reported mainly through isolated case reports and small case series [1,2]. Despite its benign nature, the chronic and progressive pigmentation can contribute to cosmetic concerns and psychosocial distress in affected patients [1]. This article presents an unusual early-onset presentation of DDD-like hyperpigmentation in a two-year-old Saudi girl, highlighting the diagnostic challenges and clinicopathological overlap encountered in pediatric pigmentary disorders.

## Case presentation

A two-year-old Saudi girl was referred to the dermatology clinic from a primary health care clinic for evaluation of asymptomatic hyperpigmentation involving the bilateral axillae and genital region. She was born at full term with an unremarkable perinatal and postnatal course. The patient’s growth parameters and developmental milestones were appropriate for her age.

Pigmentation was initially noted in the first year of age in the axillae and later involved the genital and facial areas. The lesions were asymptomatic and not associated with malodor. There was no history of topical medication use, herbal remedies, cosmetic use, or environmental exposure. None of the family members had similar skin conditions or other dermatologic diseases; however, the parents were consanguineous first-degree relatives. Systemic examination was unremarkable.

**Figure 1:**
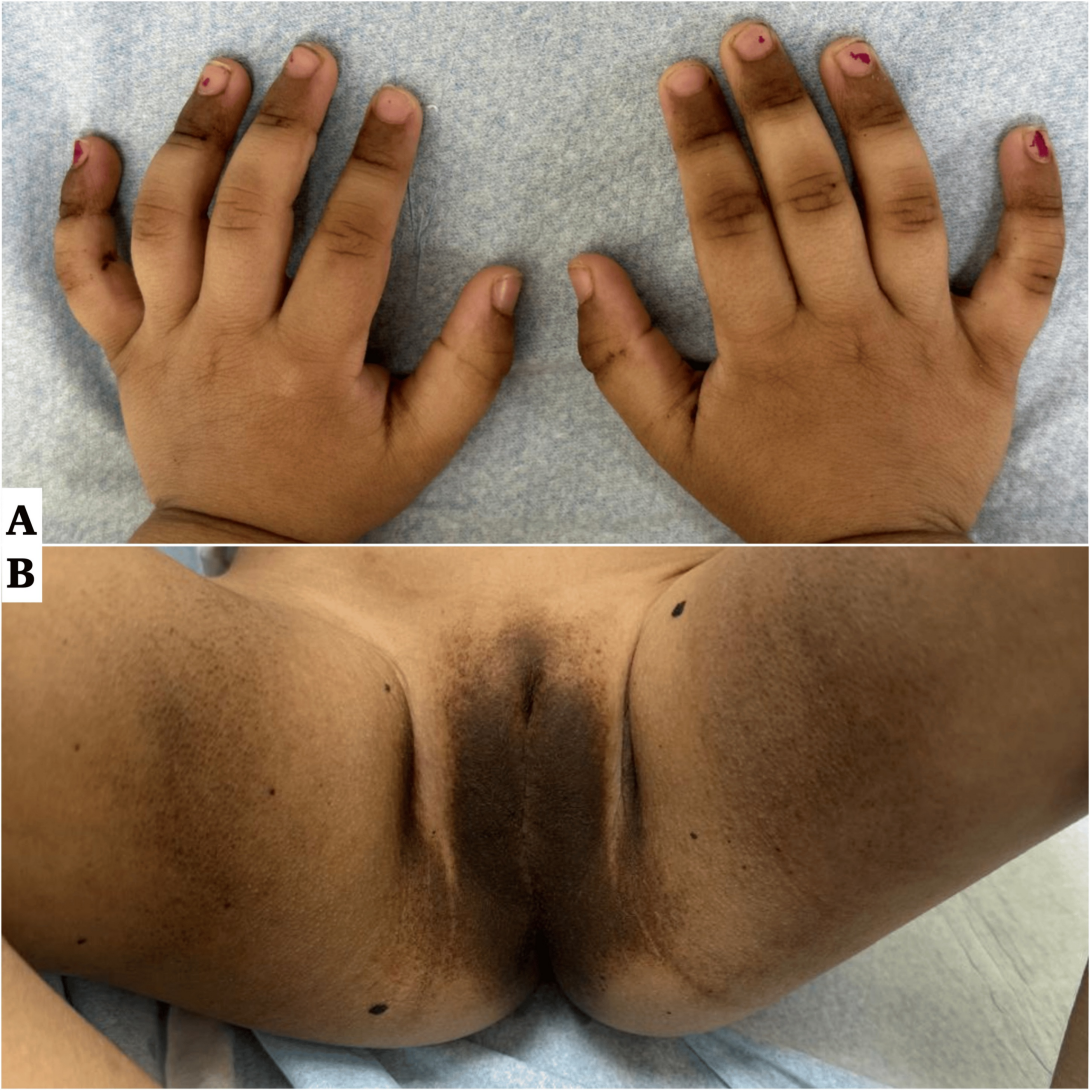
Multiple well-defined, irregular, thick, and velvety brown plaques involving the (A) dorsal fingertips, (B) and anogenital region and and medial thighs

**Figure 2:**
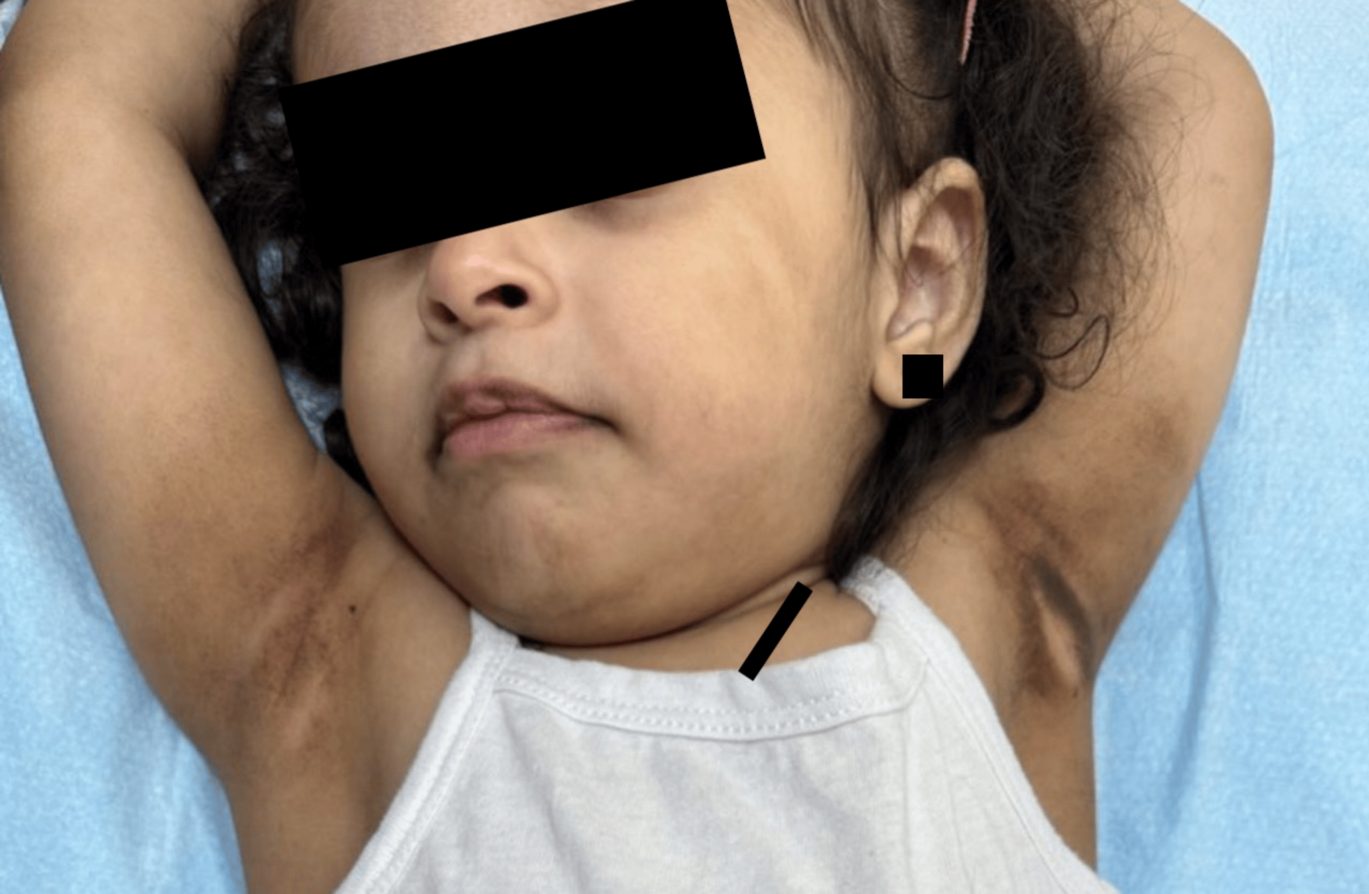
Multiple well-defined, irregular, thick, and velvety brown plaques involving the bilateral infraorbital folds, lips, axillae

Physical examination revealed multiple irregular, velvety hyperpigmented plaques involving the face and body folds (Figures 1, 2). The mucous membranes, scalp, and nails were spared.

The initial working diagnosis was acanthosis nigricans, and the patient was referred to the pediatric clinic to rule out precocious puberty due to congenital adrenal hyperplasia. Differential diagnoses included erythema dyschromicum perstans and DDD.

**Figure 3:**
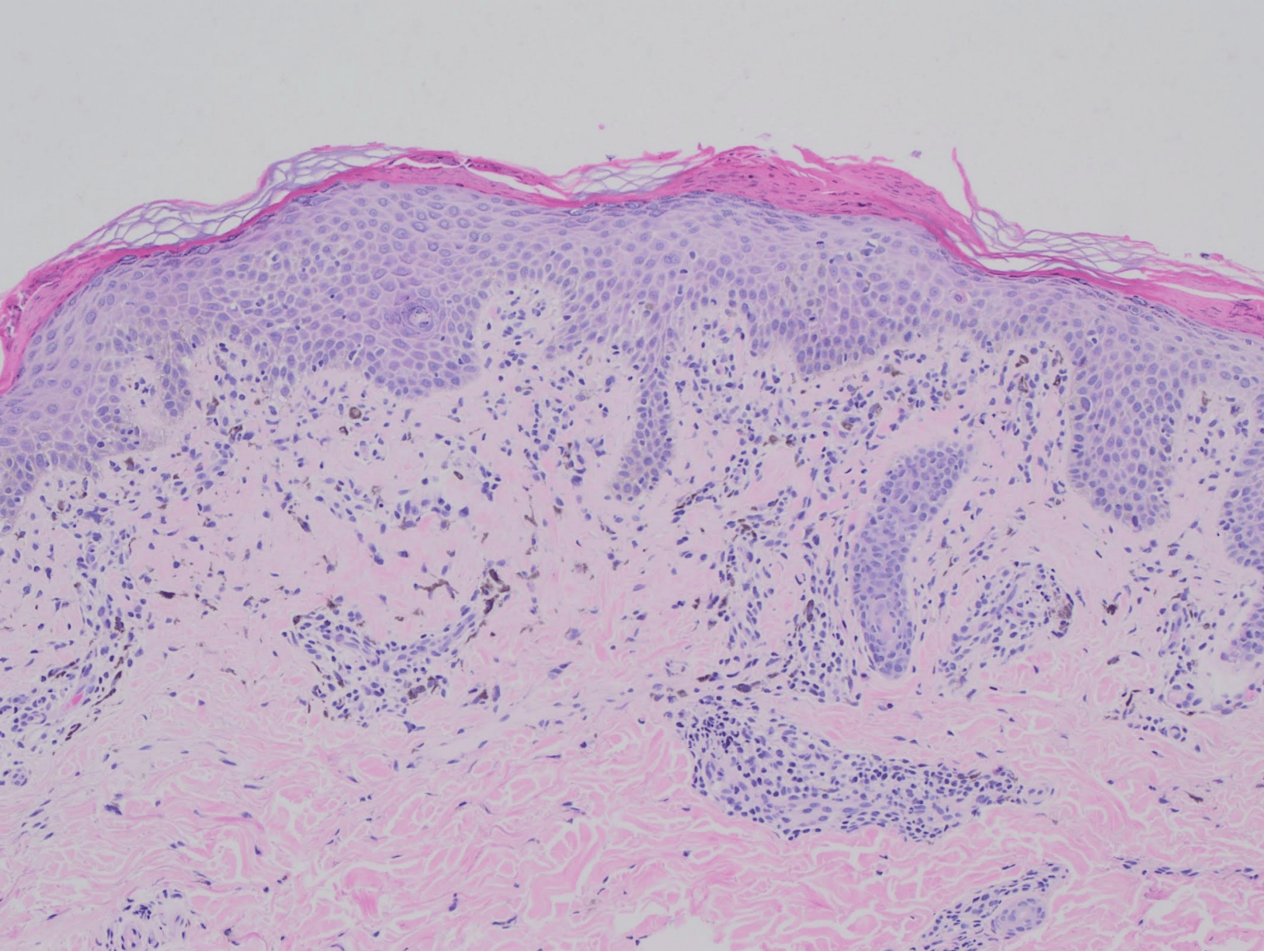
Skin punch biopsy stained with hematoxylin and eosin, showing a section extending to the superficial subcutaneous fat. The epidermis demonstrates acanthosis with elongated rete ridges and basal hypermelanosis. Original magnification ×20.

**Figure 4:**
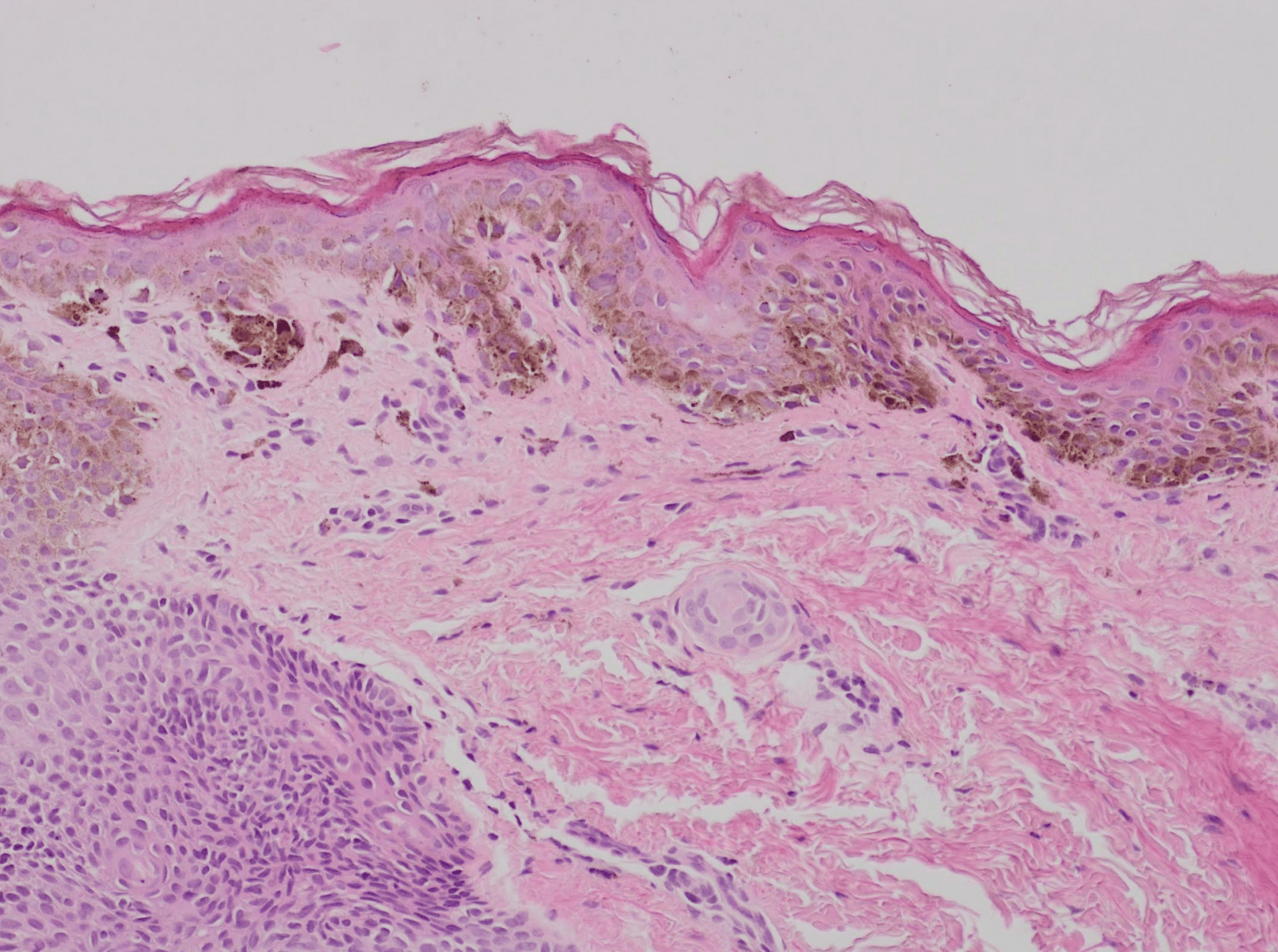
SOX10 immunohistochemical staining highlights an increased number of melanocytes with focal melanocytic nesting, composed of three-four cells, predominantly at the tips of the rete ridges. Original magnification ×10.

Histopathological examination revealed epidermal acanthosis with elongated rete ridges and prominent basal hyperpigmentation. An increased melanocyte-to-keratinocyte ratio with small benign melanocytic nests at the tips of the rete ridges was noted, without acantholysis or cytologic atypia. Scattered pigment-laden macrophages were present in the papillary dermis (Figure 3). Immunohistochemistry for SOX10 revealed an increased number of melanocytes with focal nesting. These findings supported the diagnosis of DDD-like pigmentary changes. No histopathological features supporting acanthosis nigricans were identified (Figure 4). Treatment options for DDD are limited due to the paucity of published literature. The patient was started on topical tacrolimus 0.03% ointment applied twice daily, which was well tolerated. At follow-up, the patient continued to develop new hyperpigmented lesions involving the popliteal fossae, and no change in the initial lesions despite regular application of topical tacrolimus 0.03% ointment twice daily. The patient was reassessed clinically and followed up at closer intervals with counseling regarding the chronic and progressive nature of the condition. Given the patient’s young age and limited therapeutic evidence in the literature, topical tacrolimus was continued at the same frequency as it was well tolerated without adverse effects. Patient is still being followed up to assess further disease progression and guide future management, although follow-up attendance has been inconsistent.

## Discussion

This case highlights an unusually early presentation of DDD-like flexural pigmentation. DDD pathogenesis involves mutations in the non-helical domain of keratin 5, which disrupt its interaction with the chaperone protein HSC70, thereby impairing melanosome trafficking and resulting in reticulated pigmentation. Other genes implicated include PSENEN (a component of the γ-secretase complex) and POFUT1, with PSENEN-related variants associated with hidradenitis suppurativa [2,3].

The differential diagnosis of reticulated hyperpigmentation in children includes congenital adrenal hyperplasia, which was excluded by laboratory evaluation.

Another consideration is Galli-Galli disease, which is caused by mutations in KRT5 and POGLUT1, similar to DDD. Galli-Galli disease is characterized histologically by suprabasal acantholysis, which was not observed in this case [4,5]. Reticulate acropigmentation of Kitamura is another consideration; it may present early in life, as in our case. However, it is clinically distinguished by atrophic hyperpigmented papules that coalesce into a reticulated pattern, in contrast to the thick, velvety plaques observed in our patient. It is differentiated from DDD by its initial distribution and from dyskeratosis congenita [6]. Acanthosis nigricans presents with velvety plaques but can be distinguished histologically from DDD by less pronounced elongation of the rete ridges and absence of follicular involvement [7].

Despite the distinct clinical presentation and histopathological features consistent with DDD, whole-exome sequencing did not identify pathogenic variants associated with DDD in this patient. An early-onset case of DDD was reported in India in a three-year-old girl with flexural reticulate hyperpigmentation [8]. The patient’s histopathology revealed irregular acanthosis, elongated rete ridges, and basal hyperpigmentation, supporting the diagnosis despite the absence of genetic testing. That report demonstrated that DDD-like pigmentary disease can occur in early childhood and emphasized the diagnostic value of histopathology in distinguishing it from other pigmentary disorders.

The management of DDD remains challenging, with no established standard therapy. Various treatment modalities have been reported with inconsistent efficacy. Patients should be advised to minimize friction from clothing and practice sun protection to prevent progression of hyperpigmentation. Treatment options include topical, systemic, and laser-based therapies. Topical agents include tacrolimus 0.1%, hydroquinone 5%, and corticosteroids. Topical adapalene 0.1% hasdemonstrated limited benefit and may cause irritation. Systemic retinoids, such as isotretinoin and etretinate, have generally been ineffective [9].

Laser therapy has shown promising results in the treatment of DDD. A case report described successful treatment using intense pulsed light (IPL) in a 24-year-old woman with pigmentation affecting the flexural areas, neck, and chest. Treatment parameters included a 15 × 30 mm spot size, 515- and 560-nm filters, fluences of 6 to 10 J/cm², and 10-ms pulse durations. Two IPL sessions administered over four months resulted in notable improvement, particularly at higher fluences, with no adverse effects observed during seven months of follow-up [10]. Erbium:yttrium-aluminium-garnet (YAG) ablative laser has also demonstrated efficacy, with more favorable outcomes reported after a single session. Combination therapy with isotretinoin (10 mg/day) enhanced treatment response. Q-switched Nd:YAG and fractional CO₂ lasers have also been used with less benefit [9].

## Conclusions

This case highlights a rare DDD-like pigmentary disorder in a two-year-old Saudi girl, underscoring the importance of clinical suspicion, histopathological correlation, and genetic evaluation in pediatric pigmentary disorders. To our knowledge, this is the first reported case of Dowling-Degos-like pigmentation in a patient younger than three years. Further studies are required to clarify genotype phenotype correlations in early-onset DDD and to develop targeted therapies that optimize management and improve patient quality of life.
